# An Overview of Antimicrobial Compounds from African Edible Insects and Their Associated Microbiota

**DOI:** 10.3390/antibiotics10060621

**Published:** 2021-05-22

**Authors:** Cynthia M. Mudalungu, Chrysantus M. Tanga, Segenet Kelemu, Baldwyn Torto

**Affiliations:** International Centre of Insect Physiology and Ecology (icipe), P.O. Box 30772, Nairobi GPO-00100, Kenya; ctanga@icipe.org (C.M.T.); skelemu@icipe.org (S.K.); btorto@icipe.org (B.T.)

**Keywords:** antimicrobial peptides, edible insects, microbiota, multidrug resistance, gut, small molecules

## Abstract

The need for easily biodegradable and less toxic chemicals in drug development and pest control continues to fuel the exploration and discovery of new natural molecules. Like certain plants, some insects can also respond rapidly to microbial infections by producing a plethora of immune-induced molecules that include antibacterial and antifungal peptides/polypeptides (AMPs), among other structurally diverse small molecules. The recent recognition that new natural product-derived scaffolds are urgently needed to tackle life-threatening pathogenic infections has been prompted by the health threats posed by multidrug resistance. Although many researchers have concentrated on the discovery of AMPs, surprisingly, edible insect-produced AMPs/small molecules have received little attention. This review will discuss the recent advances in the identification and bioactivity analysis of insect AMPs, with a focus on small molecules associated with the microbiota of selected African edible insects. These molecules could be used as templates for developing next-generation drugs to combat multidrug-resistant pathogens.

## 1. Introduction

Plants are the primary sources of natural products, having contributed immensely to drug discovery for the treatment of a variety of human diseases. However, the arsenal of secondary metabolites has recently expanded to include those of microbial origin associated with insects [1]. The early regime of antibiotics was boosted by the “accidental” discovery of penicillin (β-lactam), isolated from the fungus *Penicillium* sp., whose mode of action entailed targeting bacterial cell wall synthesis. Subsequent investigations in the field of antibiotic metabolites led to the discovery of many other classes of antimicrobial compounds through conventional fermentation procedures. However, the systematic emergence of resistance towards the available classes of antibiotics has increasingly posed a major challenge [2]. This has continued to justify the search for new antimicrobial metabolites, with potentially new modes of action, towards countering the problem of multi-drug resistance.

Bacteria and fungi, like many other organisms, must compete for resources by using a range of strategies, including those inflicted directly by toxins and indirectly through the activity of host immune responses, which results in changes in pathogenicity [3]. Bacterial and fungal interactions with their hosts including insects are crucial for many biological processes in agriculture, waste management, food production, and medicine [4,5].

Reviews on the nutritional value of certain insects, such as black soldier fly larvae, housefly maggot and pupae, mealworms, silkworm pupae, locusts, beetles, grasshoppers, and crickets, show that they have the potential to be used as an alternative protein source in various livestock feeds [6,7]. Additionally, insect meals can be used effectively in rearing of animals, such as poultry, to enhance animal health, intestinal morphology, and the composition of the intestinal microbiota [8], with an effect close to that of plant bioactive substances [9]. These insects’ gut microbiota comprises a diverse range of microorganisms that produce bioactive compounds that, among other things, protect their host from pathogenic assault [10]. However, little is known about these bioactive compounds, often rich in peptides and other classes of natural products.

**Figure 1:**
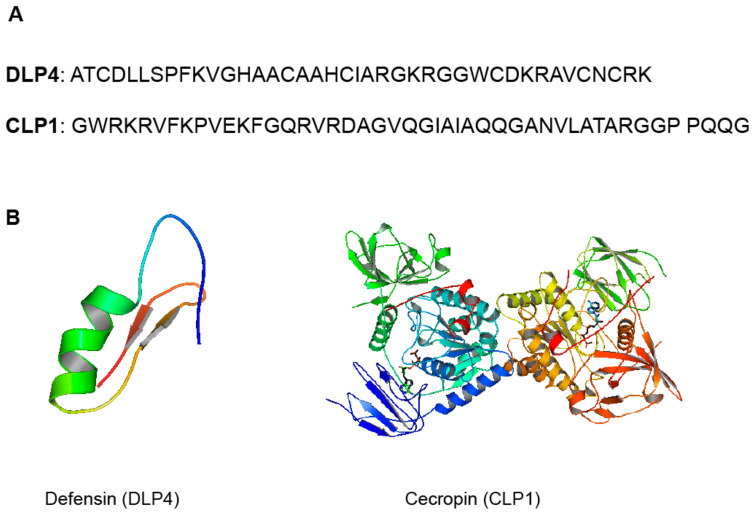
(**A**) Sequence of DLP4 (40-amino acid sequence, 4267 Da), [15] and CLP1 (46_amino acid sequence, 4840 Da) [16]. (**B**) Structural representation and molecular modeling of DLP4 (pdb code: 2nz3) and CLP1 (pdb code: 2npi). Molecular models were generated with PyMOL 2.4.1.

Antimicrobial peptides/polypeptides (AMPs), for example, are an innate component of insect immunity found in their hemolymph and have been shown to have significant biological activity against fungi, viruses, parasites, and, most importantly, antibiotic-resistant bacteria [11,12]. Antimicrobial peptides (AMPs) are small molecules that range in size from 10 to 100 amino acid (AA) residues and are produced by all living organisms. The rich diversity of insects makes them strong candidates to screen for novel sources of AMPs. The black soldier fly (BSF) *Hermetia illucens* L. (Diptera: *Stratiomyidae*), in particular, has been shown to have an extraordinary ability to live in hostile environments rich in microbial colonies, making it one of the most promising sources of AMPs [13]. Some of the AMPs identified from the BSF larvae and adult transcriptomes include defensin (44%), cecropins and lysozymes (18%), attacins (7%), and other AMPs (<5%) [13]. Structural examples of the described defensin-like peptide (DLP4) and cecropin (CLP1) from BSF are shown in Figure 1. Many other AMPs found in insects have been compiled in a mini-review by Wu et al. [14], which sheds light on their biological effects [14]. In their analysis, these authors delved mainly on the various modes of action suggested for insect AMPs. However, there is paucity of evidence to support the structure–activity relationships (SARs) and mechanisms that underpin AMP activity. There is also no information on other antimicrobial chemicals produced by insects, which is the subject of the current review, with special emphasis on edible insects.

Infections caused by resistant bacteria, on the other hand, are more difficult to treat, jeopardizing the efficacy of first-line antibiotics. As such, drugs that are more effective, more widely available, and have less toxic side effects are in high demand to treat these infections [17,18]. Thus, this review will discuss the recent advances in the identification and bioactivity of antimicrobial compounds derived from selected African edible insects and opportunities for their use as templates for developing next-generation drugs to combat multidrug-resistant pathogens. Although this review targets edible insects from Africa, in some sections, references are drawn from other parts of the world to buttress the growing demand for antibiotics.

## 2. Edible Insects in Africa and Their Microbiota

The consumption of insects (entomophagy) is an ancient and traditional practice, which has been recognized as one of the ways to alleviate hunger around the world, particularly in Latin America, Africa, and Asia. In sub-Saharan Africa, edible insects have been used as food because they are a good source of protein and essential fatty acids [19,20,21]. They are also high in micronutrients such as copper, iron, magnesium, manganese, phosphorus, selenium, and zinc, as well as vitamins such as riboflavin, pantothenic acid, biotin, folic acid, vitamin A, B complex, and C [22,23].

Africa has the most diverse collection of edible insects, with over 500 species including caterpillars (*Lepidoptera*), termites (*Isoptera*), locusts, grasshoppers and crickets (*Orthoptera*), ants, wasps and bees (*Hymenoptera*), bugs (*Heteroptera* and *Homoptera*), dragonflies (*Odonata*), flies (*Diptera*), and beetles (*Coleoptera*) [24]. As such, these different insect orders may associate with a diversity of microorganisms including bacteria and fungi.

Spore-forming bacteria and Enterobacteriaceae, for example, have previously been detected in crushed mealworms and crickets and were believed to be released from the gut [25]. Furthermore, research on cricket (*Gryllotalpa Africana*), weevil (*Rhynchophorus phoenicis*), and butterfly (*Bematistes alcinoe*) species showed that the majority of microorganisms belonged to the two bacterial genera *Bacillus* and *Staphylococcus*, with saprophytes accounting for the remainder [26].

Additionally, a recent study profiled the black soldier fly larval gut microbiota, which included bacterial and fungal communities from four different substrates (brewers’ spent grain, kitchen food waste, poultry manure, and rabbit manure). Metagenomic analysis revealed 21 bacterial and 20 fungal genera. These findings further indicated that the composition and abundance of the identified microbes differed depending upon the substrate [27]. The highly represented bacterial population in BSF larvae reared on all substrates except rabbit waste, for example, belonged to the *Dysgonomonas* genus. In contrast, the *Campylobacter* genus was more abundant in BSF larvae reared on rabbit waste than in larvae raised on the other three substrates [27]. *Pichia*, *Cyberlindnera*, and *Saccharomycecodes* were found in high concentrations in brewers’ spent grain, kitchen food waste, and rabbit manure, respectively, in the fungal community. These findings suggest that there is a large population of microorganisms that is yet to be identified, including those found in edible insects, from which new scaffolds of drugs with potentially new modes of action could be developed to target multidrug-resistant bacteria.

## 3. Multi-Drug Resistance (MDR)

The development of antibiotic resistance has continued to warrant the search for novel bioactive metabolites in the field of natural products. A contributing factor to the problem of multi-drug resistance is the widespread and uncontrolled usage of antibiotics to treat bacterial infections. Scientific research has shown that some disease-causing microbes have developed resistance against certain available classes of antibiotics. These include the “ESKAPE” organisms, i.e., *Enterococcus faecium*, *Staphylococcus aureus*, *Klebsiella pneumoniae*, *Acinetobacter baumannii*, *Pseudomonas aeruginosa*, and *Enterobacter* sp. [28]. Methicillin-resistant *Staphylococcus aureus* (MRSA) is a classic example of a notorious case of multi-drug resistance development. It has developed resistance against the major classes of antibiotics such as aminoglycosides, macrolides, tetracycline, chloramphenicol, and lincosamides [29].

Multi-drug resistance (MDR) is therefore linked to several disease treatment challenges, including prolonged time of infection in patients due to the increased spread of resistant pathogens as a result of first-line drug efficacy, high treatment costs that may lead to high morbidity and mortality rates, and the exposure of immune-compromised patients as an easy target due to decreased drug efficacy [30]. These reports encourage the exploration of alternative sources of antibiotics to overcome MDR. That begs the question of whether insects could serve as a potential source for identifying novel antibiotics.

## 4. Insects as Potential Antibiotic Producers

Insects not only perform a variety of roles in the environment, but also host a diverse community of microorganisms. The multifaceted cellular and humoral mechanisms comprise the innate immune system of an insect [31,32]. The cellular mechanism is based on phagocytosis being activated by enzymes and invading microorganisms being encapsulated by the hemolymph. The humoral response, on the other hand, is involved in the production of broad-spectrum antimicrobial peptides (AMPs), reactive oxygen or nitrogen intermediates, and complex enzymatic cascades that help to regulate hemolymph coagulation or melanization [33,34]. The presence of microorganisms invading insects causes the fat body to rapidly synthesize AMPs, which are then secreted into the hemolymph [35,36].

Previous research indicates that each insect species produces a distinct antimicrobial peptide that acts against specific microorganisms, as shown in the review by Yi et al. [11]. However, in order to boost the insect’s defense system against other pathogens, some of the peptides are expressed concurrently, encouraging synergism [36,37,38]. For example, when a cecropin (LSer-Cec6) and a defensin (LSer-Def4) from the wound maggot *Lucilia sericata* were examined together, they showed significantly increased antibacterial activity [39]. Furthermore, the amount of AMPs produced by insects varies greatly depending on the species. As such, AMPs have distinct modes of action, such as altering the electrochemical gradient at the membrane, producing reactive oxygen/nitrogen species (ROS/RNS) that cause cell death, inhibiting protein synthesis, and permeabilizing the cell membrane [38,40]. Antimicrobial peptides (AMPs) have pharmacological properties such as low molecular weight, high water solubility, broad-spectrum antimicrobial activity, and low levels of cytotoxicity [41].

## 5. The Chemistry of Microorganisms from Selected Edible Insects

In this section, chemical compounds identified in microbes found in the six selected edible insects (black soldier fly, termites, beetles, locusts, caterpillars, and crickets) are highlighted. Some of these chemicals have been reported to have antibacterial, antifungal, antimalarial, anti-inflammatory, and cytotoxic activities.

### 5.1. Black Soldier Fly Hermetia Illucens (Diptera: Stratiomyidae)

The gut of the black soldier fly (BSF) larvae has been shown to harbor beneficial microbes and fungi that also control pathogens [42]. Thus, the microbes linked to it are a good target for the discovery of new antimicrobial compounds of pharmaceutical relevance. In some studies, the *Pichia* genus was found to be related to the larvae fed on vegetable waste, whereas *Trichosporon*, *Rhodotorula*, and *Geotrichum* were the most abundant genera in the larvae fed on chicken feed only [42]. Regarding the production of AMPs, it is highly conserved between insects and may only vary between species depending on their respective habitats [43]. In BSF larvae, AMPs have been classified as defensins (cysteine-rich peptides), cecropins (α-helical peptides), attacins (glycine-rich peptides/proteins), and diptericins (a family of related glycine-rich antibacterial peptides), as well as lipids (hexanedioic acid) [43,44]. In a previous analysis, scores that predicted the biological activity of unknown peptides in the transcriptomes of BSF larvae and adults were determined using different algorithm in in silico tests. Biological activities that were predicted included antimicrobial, anticancer, antiviral, and antifungal properties [13]. Follow-up in vitro studies are yet to be carried out to validate these findings.

The general mechanisms of action of AMPs have been investigated in a few studies [45,46]. For example, Park et al. [47], demonstrated that *H. illucens* larvae extracts have antibacterial properties against Gram-positive *S. aureus*, methicillin-resistant *S. aureus* (MRSA), and Gram-negative *Pseudomonas aeruginosa* [47]. The methanol extract of BSF was found to inhibit the growth of *K. pneumoniae*, *Neisseria gonorrhoeae*, and *Shigella sonnei* bacteria, whereas the extract elicited no antibacterial effects against the bacteria *B. subtilis*, *Streptococcus mutans*, and *Sarcina lutea* [48].

The expression and characterization of stomoxynZH1 (encoded by a 189-basepair gene) with antimicrobial activity against *Escherichia coli* and *S. aureus* is one of two recent promising results involving AMPs from *H. illucens* [49]. In addition, DLP4, a novel AMP isolated and characterized from *H. illucens* hemolymph larvae, showed potent activity against MRSA and methicillin-susceptible *S. aureus* (MSSA) [15].

**Figure 2:**
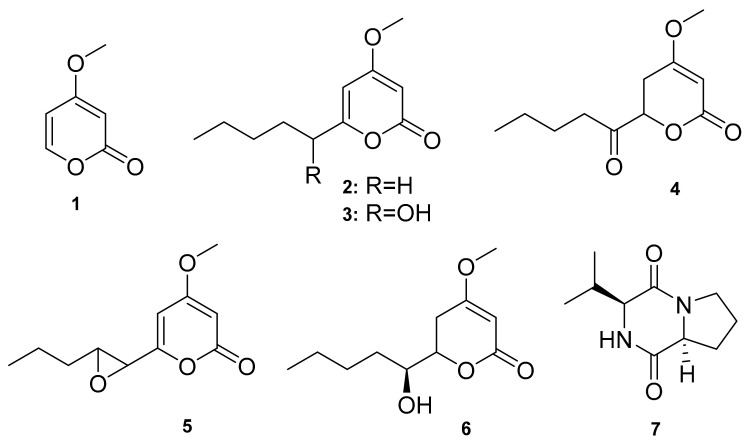
Small molecules isolated from the midgut of BSF larvae.

Furthermore, secondary metabolites from *Chrysosporium multifidum* broth extract, a fungus isolated from the midgut of BSF larvae fed on fresh unsterilized chicken guano, have been described in the literature. As shown below, these molecules include six pyrone derivatives (**1**–**6**) and one diketopiperazine (**7**) (Figure 2). The epoxy moiety containing α-pyrone (**5**) was found to have moderate antibacterial activity against 43,300 ATCC strains of methicillin-resistant *Staphylococcus aureus* (MRSA) [10]. Thus, AMPs and small molecules from *H. illucens* are still understudied, despite the fact that they have strong antibacterial properties. So far, only one fungal strain has been targeted within the rich microbiota of *H. illucens*, with no reports on the antifungal and cytotoxic effects of the identified molecules.

### 5.2. Termites (Isoptera)

Termites are recognized as beneficial insects in agriculture, entomotherapy, and the environment. Their ability to act as mediators of the process of decomposing plant organic matter and as influential agents in soil formation, particularly in tropical forests, is one of their important ecological roles [50]. The diversity of termite gut communities is astounding, but the function of each group of symbionts is poorly understood. Hemicellulose-degrading bacteria, lignolytic bacteria, cellulolytic bacteria, aromatic compound-degrading bacteria, and nitrogen-fixing bacteria are among the primary termite intestinal microorganisms [51,52,53,54,55,56,57,58,59]. According to research, the most abundant bacteria found in both higher and lower termites are strict aerobes or facultative anaerobes. There is also evidence that the most abundant bacteria in termite guts belong to the *Staphylococcus* and *Bacillus* genera [60,61]. Moreover, research has previously discovered a link between the major gut bacteria and the termite family (see Table 1) [62].

**Figure 3:**
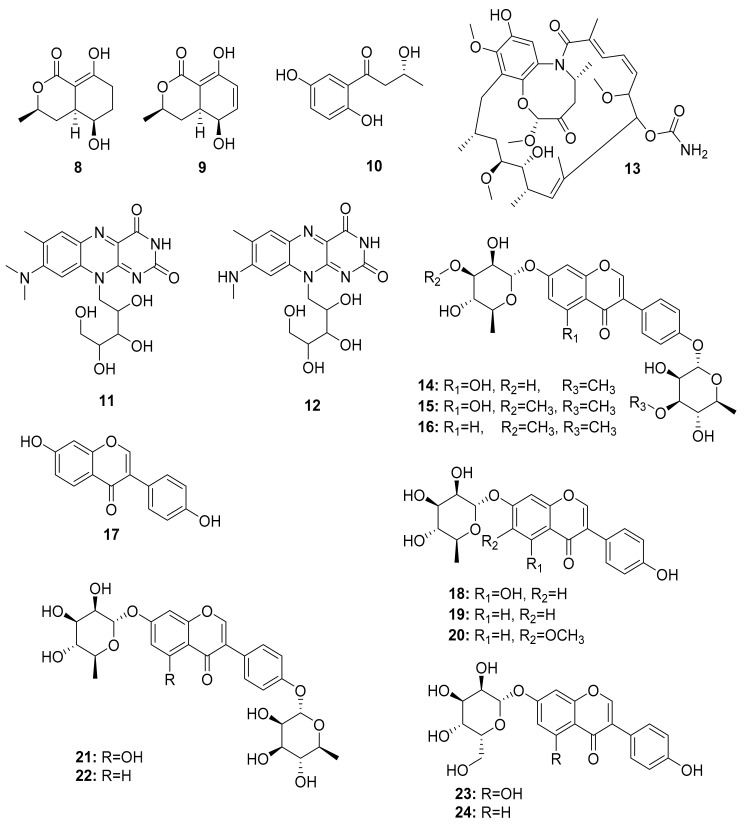
Small molecules isolated from fungi related to *Odontotermes formosanus* and *Streptomyces* strains.

Additional studies have shown that *Streptomyces* strains isolated from termites had significantly higher inhibition activity against Gram-negative bacteria than soil isolates [64]. Chemical analysis of termite-associating microbes, in particular fungi from *O. formosanus*, revealed three small molecules named 5-hydroxyramulosin (**8**), biatriosporin M (**9**) from the *Pleosporales* sp. BYCDW4, and 1-(2,5-dihydroxyphenyl)-3-hydroxybutan-1-one (**10**) from the *Microdiplodia* sp. BYCDW8. Investigation of their biological activity indicated that compound (**10**) had moderate inhibitory activity against *B. subtilis* and *S. aureus* [65]. *Streptomyces davaonensis* YH01, isolated from the body surface of the queen of the termite *O. formosanus*, demonstrated antibacterial activity. Roseoflavin (**11**) and 8-methylamino-8-demethyl-d-riboflavin (**12**), which exhibited antibacterial activities, were discovered in a subsequent study on the same strain [66]. Their absolute stereochemistry, however, is yet to be assigned (Figure 3).

**Figure 4:**
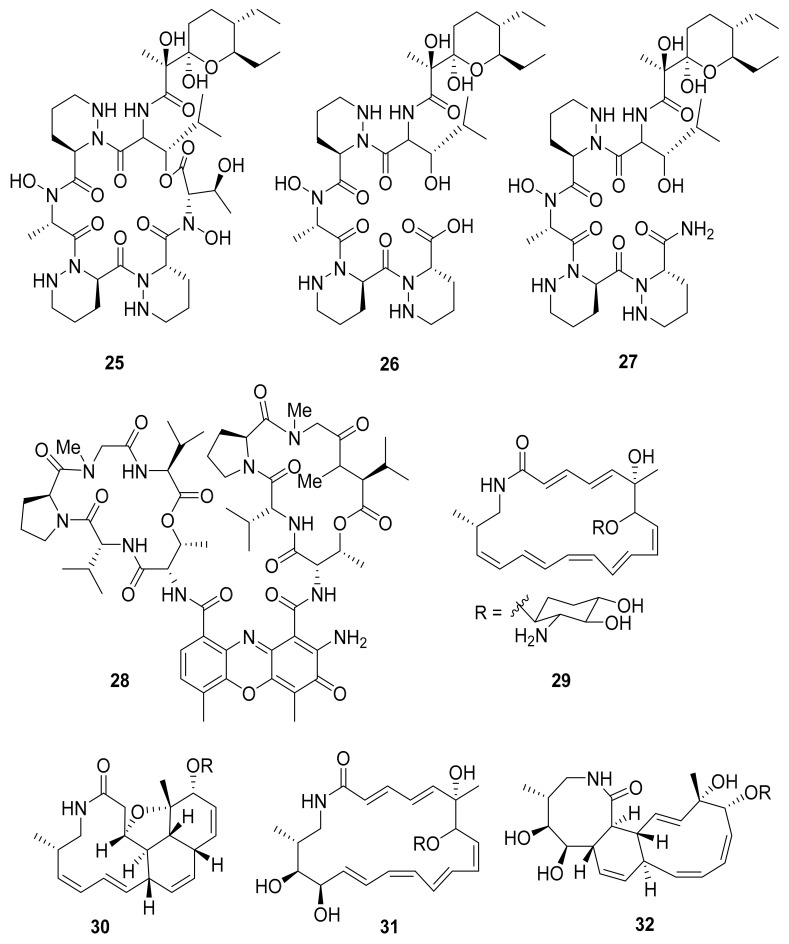
Peptides from *Streptomyces* strains and macrolactams from *Amycolatopsis* sp. associated with *Macrotermes natalensis* termites.

**Figure 5:**
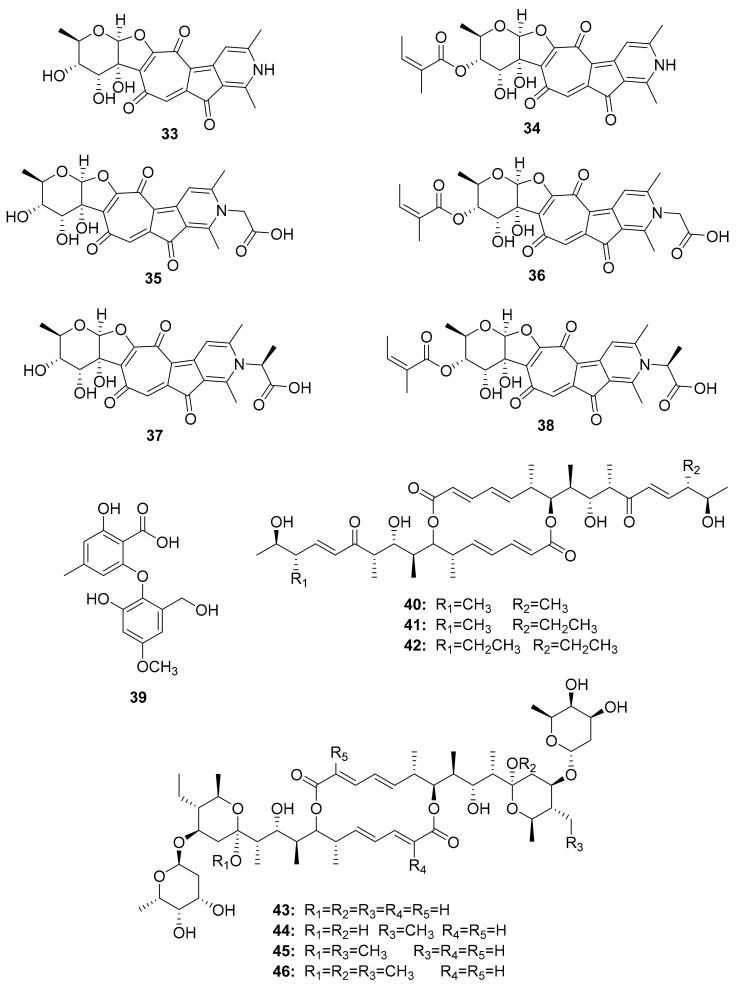
Tropolone derivatives, small molecules obtained after co-culture experiments, and efomycin-related compounds.

Several other studies have shown that Actinobacteria in association with *Macrotermes natalensis* (a fungus-growing termite species) led to the isolation of natalamycin (**13**) from *Streptomyces* sp. M56, a new antifungal geldanamycin derivative [67]. Termisoflavones A–C (**14**–**16**) and eight isoflavonoid molecules (**17**–**24**) have been reported from *Streptomyces* sp. RB1 [68]. In addition, the structures of the complex nonribosomal peptide synthetase–polyketide synthase (NRPS/PKS) hybrid depsipeptides dentigerumycins B–D (**25**–**27**) were characterized from *Streptomyces* sp. M41 [69]; actinomycin D (**28**) was isolated from *Streptomyces* sp. RB94 [70]. The glycosylated polyketide macrolactams macrotermycin A–D (**29**–**32**) have also been described in *Amycolatopsis* sp. M39 [71], and a group of tropolone derivatives, rubterolone A–F (**33**–**38,**) were found in *Actinomadura* sp. RB29/5-2 [70,72]. Co-culture studies of *Streptomyces* sp. RB108 with *Pleosporales* sp. also yielded the PKS-derived barceloneic acid A (**39**), which acts as a farnesyl-protein transferase inhibitor (Figure 4 and Figure 5) [70,73]. Other than (**19**) and (**24**), no antimicrobial activity was found for termisoflavones and isoflavanoid compounds (see Table 2). At a cisplatin dose of 25 μM, the two compounds reduced cisplatin-induced kidney cell damage to 80% of the control value [68].

Further research on *Streptomyces* sp. M56 resulted in the identification of two new compounds with an unsaturated enone moiety, named efomycins K (**40**) and L (**41**), in addition to the well-known efomycin M (**42**), a potent and specific inhibitor of selectin [74,75,76]. Subsequently, five known and structurally related hemiketal derivatives—efomycin G (**43**) [77], elaiophylin (**44**) [78], 11-*O*-methylelaiophylin (**45**) [78], and 11,11′-*O*-dimethylelaiophylin (**46**)—were isolated [79,80].

**Figure 6:**
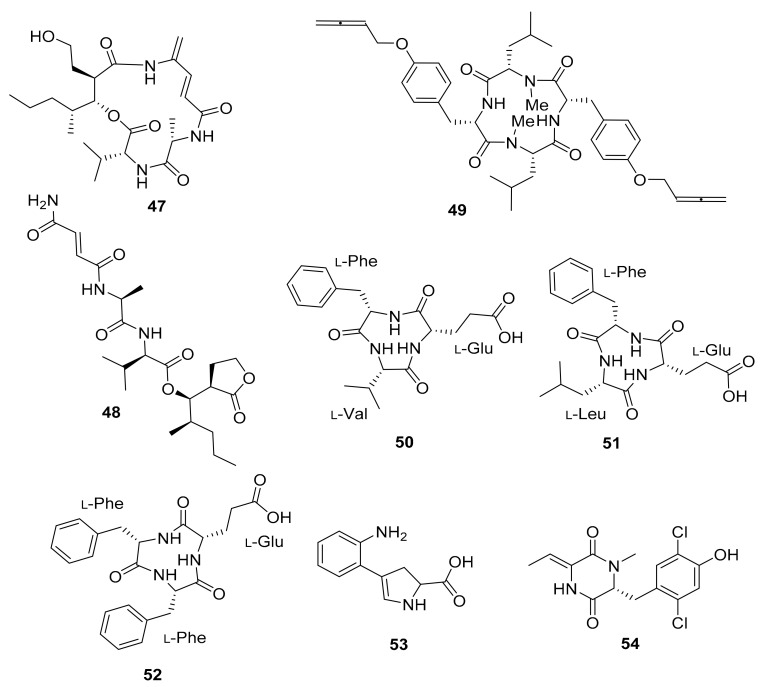
Peptides isolated from the microbiota of *Microtermes* sp. and *Macrotermes natalensis* termites.

The investigation of *Streptomyces* sp. MspM5 found in a South African fungus-growing termite, *Microtermes* species, led to the discovery of two novel PKS/NRPS pathway peptides. The peptides, known as microtermolide A (**47**) and B (**48**), exhibited no antibacterial or antifungal activity [84]. *Pseudoxylaria* sp. X802, which evolved from *Microtermes* sp., produces a number of antimicrobial compounds. Pseudoxyallemycin B (**49**) is one of the isolated antibacterial peptides, with a rare and chemically accessible allene moiety (Figure 6) [85].

Three new cyclic tripeptides named natalenamides A–C (**50**–**52**) were isolated from the termite-associated *Actinomadura* sp. RB99 isolated from the fungus-growing termite *Macrotermes natalensis*. Compounds **50** and **51** exhibited weak cytotoxicity in HepG2 and HeLa/A549 cells, whereas compound **52** inhibited IBMX-mediated melanin synthesis in a dose-dependent manner [86]. Furthermore, co-cultivation experiments with *Actinomadura* sp. RB29 and *Trichoderma* sp. led to the discovery of antifungal compounds such as banegasine (**53**) and cyclo(*NMe*-l-*3,5*-dichlorotyrosine-Dhb) (**54**) [70].

**Figure 7:**
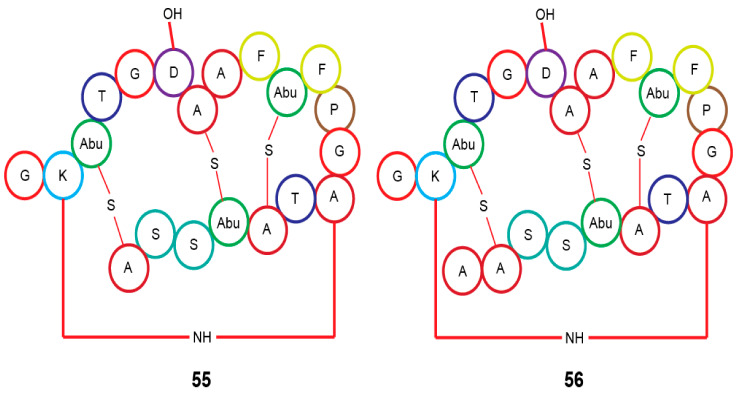
Proposed lanthipeptides from *Actinomadura* sp. RB29 linked to *Macrotermes natalensis* termites. Abu, 2-aminobutyric acid.

In silico analysis of the genome of *Actinomadura* sp. RB29 from *Macrotermes natalensis*, using antiSMASH and tandem MS^2^ data submitted to Global Natural Product Social Molecular Networking (GNPS), and subsequent RiPPquest processing revealed the presence of two lanthipeptides with proposed structures of rubrominin A (**55**) and B (**56**) [70]. Their gene cluster resembles that of cinnamycin (Figure 7) [100,101,102]. Except for the Actinobacteria microorganisms, the termite family has a large range of microbiota that has not been thoroughly investigated in terms of their chemical capacity as presented here. The bulk of their chemical structures consists of peptides and isoflavonoids, which makes them peculiar.

### 5.3. Beetles (Coleoptera)

Beetles are generally known to benefit the environment (nutrient recyclers, pollinators), but a significant portion of them are pests of economically important crops and storage products [103]. Bark beetles, in particular the southern pine beetle (*Dendroctonus frontalis*), are damaging to trees [104]. They have a symbiotic relationship with the fungus *Entomocorticium* sp. A, which serves as food for the beetle larvae [105].

Recent research has revealed a link between various beetle species and four bacterial phyla, *Proteobacteria*, *Firmicutes*, *Actinobacteria*, and *Bacteroidetes*, as well as three fungal phyla, *Ascomycota*, *Zygomycota*, and *Basidiomycota*. These microbial communities were discovered to differ depending on the beetle host, individual organism, and environment [106].

Actinobacteria, particularly *Streptomyces*, have been described as having a chemical defense mechanism that produces antimicrobial compounds that aid in the fight against infectious disease. Mycangimycin (**57**), frontalamide A (**58**), and frontalamide B (**59**), for example, were isolated from a *Streptomyces* strain that was symbiotically associated with the southern pine beetle (*Dendroctonus frontalis*). Mycangimycin inhibits the beetles’ antagonistic fungus *Ophiostoma minus* and has potent antimalarial activity. Frontalamide A and frontalamide B have antifungal properties [87,88].

Nakashima et al. studied the fungal strain *Fusarium* sp. from the ambrosia beetle *Euwalecea validus* in the early 1980s. Its culture extract contained two antifungal compounds, cerulenin (**60**) and the nortriterpenoid helvolic acid (**61**), which inhibited the growth of mold fungi and are thought to suppress bacterial contaminations [89]. Chemical analysis of rove beetles (*Paederus* sp.) yielded a complex polyketide pederin (**62**) molecule from an endosymbiotic *Pseudomonas* sp. [49,50,51,52] that showed toxicity against predators such as wolf spiders in a study by Kellner and Dettner [90].

**Figure 8:**
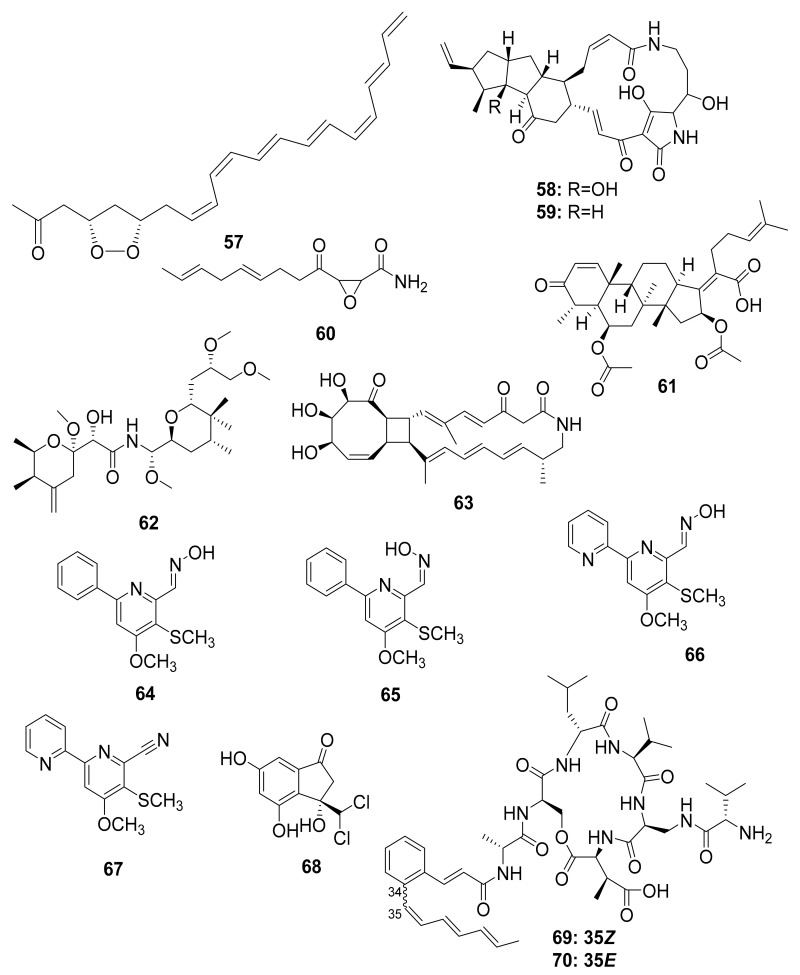
Chemical diversity in the microbiota of certain beetles.

**Figure 9:**
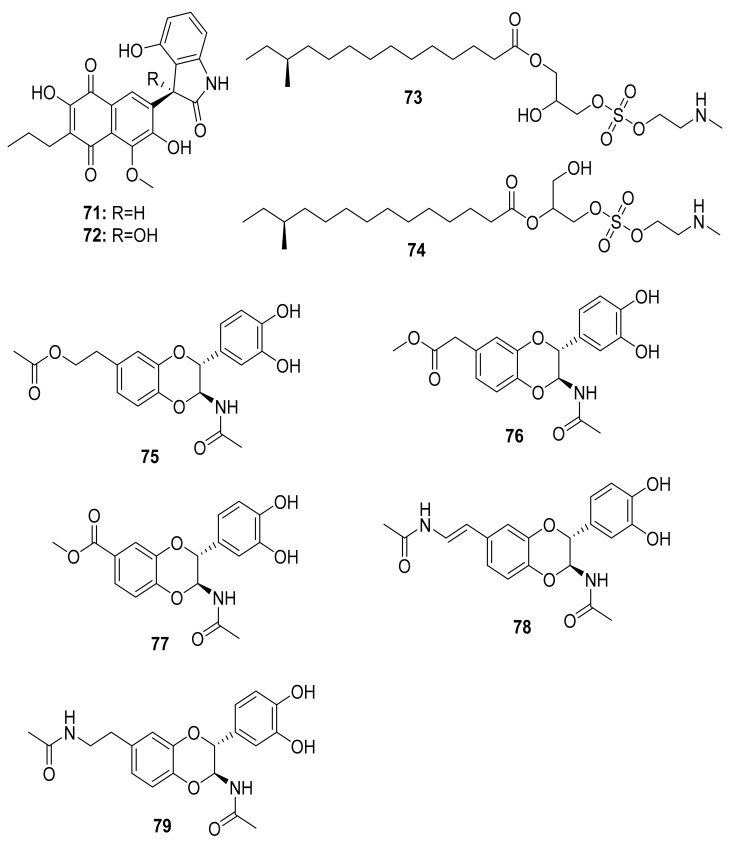
Chemical structures of compounds isolated from microorganisms linked to the dung beetles.

The soil-dwelling Korean dung beetle (*Copris tripartitus*) has previously been chemically studied and found to contain a diverse array of Actinobacteria. Their distinct metabolomic profiles resulted in the isolation of tripartilactam (**63**), a new tricyclic macrolactam that lacks antimicrobial activity but acts as a Na^+^/K^+^ ATPase inhibitor [91,92]. Follow-up studies by the same group revealed phenylpyridines, coprismycin A–B (**64**–**65**) exhibiting neuroprotective effects, dipyridines, collismycin A (**66**), SF2738D (**67**), and a dichlorinated indanone tripartin (**68**), an inhibitor of the histone H3 lysine 9 demethylase KDM4 in HeLa cells [91,93]. More recently, new cyclic heptapeptides named coprisamides A–B (**69**–**70**) were isolated from a *Streptomyces* strain found in the gut of *C. tripartitus*, demonstrating significant activity for the induction of quinone reductase (Figure 8) [94]. About a year later, the naphthoquinone–oxindole alkaloids Coprisidins A (**71**) and B (**72**) were isolated from the same *Streptomyces* strain previously studied by Um et al. [95]. Coprisidin A (**71**) was shown to inhibit the action of Na^+^/K^+^ ATPase, whereas Coprisidin B (**72**) showed induction of NAD(P)H:quinone oxidoreductase 1 (Figure 9) [95].

The antibacterial molecules lenzimycins A (**73**) and B (**74**) were isolated from the *Brevibacillus* sp. PTH23, associating with the dung beetle *Onthophagus lenzii*. These molecules were also discovered to be effective in activating a reporter system designed to detect bacterial cell envelope stress [96]. Furthermore, the study of biological agents from the entire body of the dung beetle *Catharsius molossus* resulted in the discovery of three new *N*-acetyldopamine dimers, molossusamide A–C (**75**–**77**), in addition to other known compounds, i.e., *cis*-2-(3′,4′-dihydroxyphenyl)-3-acetylamino-7-(*N*-acetyl-2″-amino-ethylene)-1,4-benzodioxane (**78**) and *trans*-2-(3′,4′-dihydroxyphenyl)-3-acetyl-amino-7-(*N*-acetyl-2″-aminoethyl)-1,4-benzodioxane (**79**) [97]. With the exception of **78**, which exhibited inhibitory effects against COX-1 and COX-2, these molecules lacked biological activity against the tested organisms (see Table 2 and Figure 9).

### 5.4. Locusts (Orthoptera: Acrididae)

Locusts, like other insects, have bacterial cells both inside and outside their bodies, such as on their cuticles. With respect to other locust species, the bacterial composition of the desert locust (*Schistocerca gregaria*) gut has been extensively described [107]. For example, the bacteria in the hindgut of the desert locust were discovered to be relatively simple, consisting of members of the families *Enterobacteriaceae* (including *Enterobacter* and *Klebsiella*) and *Enterocococeaea* [108]. This study followed previous research that found gut bacterial flora in *S. gregaria* that included *Escherichia coli*, *Enterobacter liquefaciens*, *Klebsiella*, *Pneumoniae*, and *Enterobacter cloacae* [109,110]. The lack of complexity in the desert locust’s gut microbiota could be attributed to its simple structure and short throughput time.

Despite numerous studies on the desert locust, there is little documented knowledge about the antimicrobial compounds it produces. Using gas chromatography–mass spectrometry (GC–MS), researchers recently investigated how ingested phytosterols are metabolized and biotransformed into other derivatives such as desmosterol, (3β, 5α) cholesta-8, 14, 24-trien-3-ol, 4, 4-dimethyl, (3β, 20R) cholesta-5, 24-dien-3, 20-diol, present as the dealkylated products of lanosterol in the gut [111]. However, the biological activity of these sterols against multidrug-resistant pathogens is yet to be investigated.

### 5.5. Caterpillars (Lepidoptera)

Caterpillars are among the most popular, consumed, and economically valuable edible insects in the tropics, because they have high protein and fat content [21]. They are mostly found in tropical rainforests. There are over 130 edible caterpillar species consumed in Africa alone, compared to nearly 400 species worldwide [112]. Despite their enormous contribution to global food security, little is known about their ecological relevance [113], associated microbiota, and antimicrobial potential. Caterpillars, unlike other living organisms, do not have a gut microbiota, demonstrating their freedom from symbionts [114].

**Figure 10:**
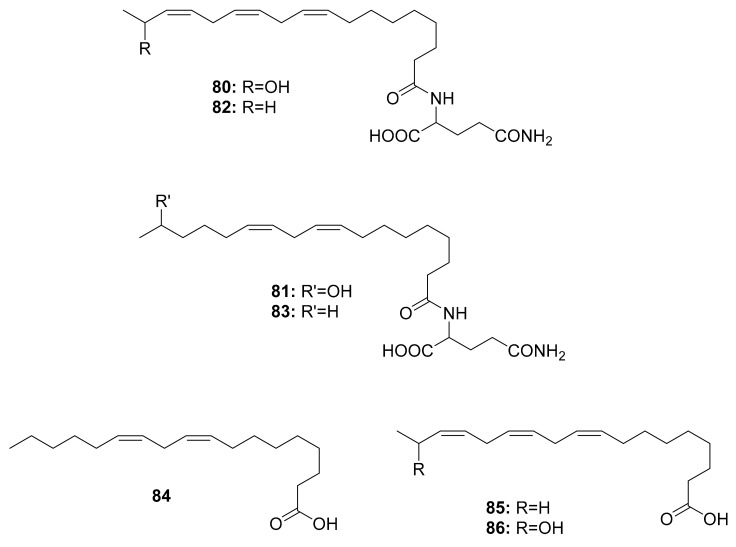
Compounds isolated from different caterpillar species.

So far, volicitin (**80**), *N*-(17-hydroxy-linoleoyl)-l-glutamine (**81**), *N*-linolenoyl-l-glutamine (**82**), *N*-linoleoyl-l-glutamine (**83**) and volicitin-related compounds (**84**), linolenic acid (**85**), and the 17-hydroxylinolenic acid (**86**) (Figure 10) have been discovered in the oral secretions of three Noctuidae species, i.e., *Helicoverpa armigera*, *Mythimna separata*, and *Spodoptera litura*, as well as one Sphingidae species, *Agrius convolvuli* [98]. The structure–activity relationships of volicitin-related compounds were investigated to determine the elicitor activity of volatiles from corn seedlings, revealing that chirality at C-17 in the linolenic acid chain had no effect on bioactivity. However, it was discovered that the l-glutamine moiety was more important than the hydroxyl moiety [115]. Derivatizing these molecules can lead to a wide variety of agricultural applications, such as identifying volatile components that can be used in pest control. Interestingly, there is no evidence of these compounds being associated with the gut microbiota of caterpillars.

### 5.6. Crickets (Orthoptera)

**Figure 11:**
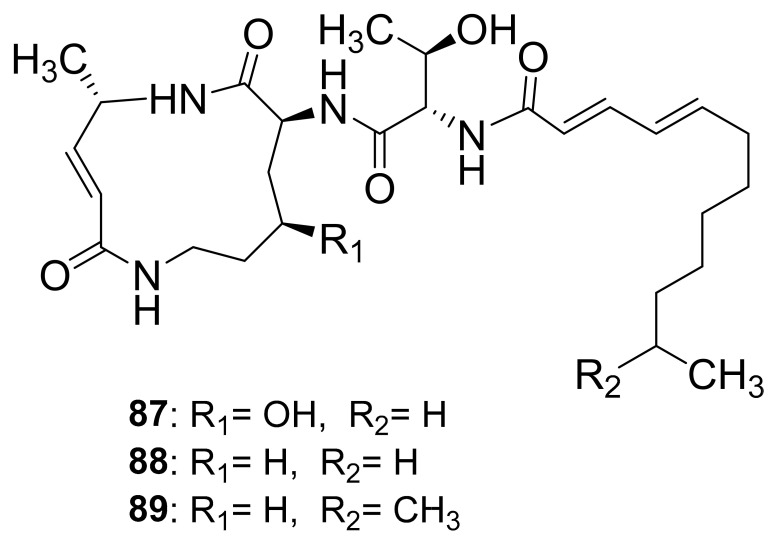
Compounds obtained from crickets infected with the *Photorhabdus asymbiotica* bacterium.

Crickets have traditionally been used as a source of medicine for a variety of ailments. In Korea, for example, the mole cricket *Gryllotalpa Africana* (*Gryllotalpidae*) is used to treat urine retention, urolithiasis, edema, lymphangitis, and furuncles [116]. In Latin America, the house cricket *Acheta domesticus* (*Gryllidae*) is used to treat scabies, asthma, eczema, lithiasis, earache, oliguresis, rheumatism, urine retention, urinary incontinence, and ophthalmological problems [117]. The crickets *Paragryllus temulentus (Gryllidae)* and *Gryllus assimilis (Gryllidae)* are both useful in the treatment of rheumatism and warts. Interestingly, bacteria from the *Citrobacter*, *Klebsiella*, *Yersinia*, *Bacteroides*, and *Fusobacterium* genera were found in the gastrointestinal tract of the house cricket *Acheta domestica* [118]. The *Photorhabdus asymbiotica* bacterium is known to have a two-part life cycle that colonizes the intestines of entomophagous nematodes and necessitates adaptation to both symbiotic and pathogenic phases [119]. The infection of live crickets with the *Photorhabdus asymbiotica* bacterium resulted in the isolation and characterization of glidobactin A (**87**), luminmycin A (**88**), and luminmycin D (**89**). Only compound (**89**) was found to be cytotoxic to human pancreatic cells and inhibited proteasome activity (Figure 11) [99]. The macrolactam center of these molecules consists of nonribosomal peptide synthetase modules catalyzing the condensation of 4-hydroxylysine, l-alanine, and malonyl-CoA [120]. Additionally, there is a threonine moiety connecting it to the fatty acid side chain. In this regard, from the perspective of structure–activity relationships, the hydroxyl group on the threonine side chain and the R_1_ positions can be targeted as accessible sites for modifying the molecule to potentially enhance its biological activities.

## 6. Future Perspectives

Insects thrive in Africa’s tropical climate. However, this review reveals that very little research has been done on the chemistry of edible insects and their microbiota in Africa. With the current knowledge, it is imperative to advance studies to a higher level, by developing various methods to isolate and characterize new AMPs and small molecules. This may involve varying the habitats in which the insects are reared to stimulate different microbiota, which may increase the likelihood of discovering new pharmacologically relevant metabolites. It is also possible to screen different life stages because insects may live in different habitats, aquatic and/or terrestrial; hence, their potential association with different microorganisms. Moreover, derivatization can be facilitated by using known chemical structures as a template. Aside from that, the identified molecules can be tested in binary or ternary mixtures to increase their synergistic/additive effectiveness against multidrug-resistant species. Bioengineering and biotransformation of species and scaffolds, respectively, may also enhance the observed biological activity. In addition, insights into how these molecules are synthesized by various organisms would be useful to identify the enzymes involved. These studies should therefore help to clarify what function these microorganisms play in their hosts and pave the way for the production of antimicrobial agents.

## 7. Concluding Remarks

This review has shown that edible insects are a good source of novel antimicrobial peptides and compounds that can be screened against multidrug-resistant pathogens. StomoxynZH1 and DLP4 are among the AMPs found in the BSF with potent antibacterial activity. The majority of isolated microorganisms from which small molecules have been identified were sourced from the insect’s gut. Overall, termite-related microorganisms are the most researched in terms of chemical diversity. In comparison to fungal strains, bacterial strains, especially Actinobacteria, have been extensively studied. As it is evident, *Streptomyces* yielded 25 (52%) of the 48 molecules isolated from termites, while 13 (27%) were isolated from *Actinomadura* species. In terms of the chemistry of BSF-associated microorganisms, only one fungal strain has been investigated so far and identified to contain primarily antibacterial α-pyrone molecules. In addition, about 29% of the small molecules characterized herein were obtained from the gut microbiota of beetles. Apart from sterols that have been identified in locusts, there is little knowledge on antimicrobial compounds that they may produce. Caterpillars, on the other hand, contain mainly linolenic acid-derived molecules in their oral secretions. Although the number of studies documenting antimicrobial peptides and small molecules is on the rise, the microbiota of edible insects still remains an understudied topic.

## Figures and Tables

**Table 1:** Classification of microorganisms found in termites.

Termite Family/Genera	Related Microbe Genera	Source	Ref
Formosan termite: *Coptotermes formosanus*	*Serratia marcescens*, *Enterobacter aerogens*, *Enterobacter cloacae*, and *Citrobacter farmeri*.	Hindgut	[63]
*Odontotermes formosanus*	*Bacillus* sp., *Citrobacter freundii*, *Pseudomonas aeruginosa Salmonella entrica*, *Enterococcus casseliflavus*, *Staphylococcus gallinarum*, and *Serratia marcescens*.	Gut	[52]
*Mastotermes darwiniensis*	*Streptococcus* sp.	Gut	[62]
*Cryptotermes primus*	*Streptococcus* sp.	Gut	[62]
*Rhinotermitidae* species: (*Heterotermes ferox*, *Coptotermes acinaciformis*, *C. lacteus*, and *Schedorhinotermes intermedius intermedius*)	*Enterobacter* sp.	Gut	[62]
*Termitidae* species (*Nasutitermes exitiosus*, *N. graveolus*, *N. walkeri*)	*Staphylococcus* sp.	Gut	[62]

**Table 2:** Small molecules from selected edible insects.

Insect	Origin, Producer Organism	Compounds	Biological Activity	Ref.
Black Soldier Fly	*Chrysosporium multifidum*	Pyrone derivatives (**1**–**6**), Diketopiperazine (**7**)	Antibacterial	[10]
Termites	*Pleosporales* sp. BYCDW4	5-Hydroxyramulosin (**8**), biatriosporin M (**9**)	Antifungal -	[65]
	*Microdiplodia* sp. BYCDW8	1-(2,5-Dihydroxyphenyl)-3-hydroxybutan-1-one (**10**)	Antibacterial	[65]
	*Streptomyces davaonensis* YH01	Roseoflavin (**11**), 8-methylamino-8-demethyl-d-riboflavin (**12**)	Antibacterial	[66]
	*Streptomyces* sp. M56	Natalamycin (**13**), Efomycins K (**40**), and L (**41**), Efomycin M (**42**), Efomycin G (**43**), Elaiophylin (**44**), 11-*O*-methylelaiophylin (**45**), 11,11′-*O*-dimethylelaiophylin (**46**)	Antifungal Antifungal Selectin inhibitor	[67,74,75,76,77,78,79,80]
	*Streptomyces* sp. RB1	Termisoflavones A–C (**14**–**16**), Isoflavanoids (**17**–**24**)	Cisplatin-induced cytotoxity	[68]
	*Streptomyces* sp. M41	Dentigerumycins B–D (**25**–**27**)	-	[69]
	*Streptomyces* sp. RB94	Actinomycin D (**28**)	Actibacterial, antitumor	[70,81,82]
	*Amycolatopsis* sp. M39	Macrotermycin A–D (**29**–**32**)	Antibacterial, antifungal	[71]
	*Actinomadura* sp. RB29/5-2	Rubterolone A–F (**33**–**38**)	anti-inflammatory activity	[70,72,83]
	Co-culture: *Streptomyces* sp. RB108 with *Pleosporales* sp.	Barceloneic acid A (**39**)	Farnesyl-protein transferase inhibitor	[70,73]
	*Streptomyces* sp. MspM5	Microtermolide A–B (**47**–**48**)	-	[84]
	*Pseudoxylaria* sp. X802	Pseudoxyallemycin B (**49**)	Antibacterial	[85]
	*Actinomadura* sp. RB99	Natalenamides A–C (**50**–**52**)	Cytotoxic, anti-inflammatory activity	[86]
	Co-culture: *Actinomadura* sp. RB29 and *Trichoderma*	Banegasine (**53**), Cyclo(*NMe*-l-*3,5*-dichlorotyrosine-Dhb (**54**)	Antifungal	[70]
	*Actinomadura* sp. RB29	Rubrominin A–B (**55**–**56**)	-	[70]
Beetles	Pine beetles, *Streptomyces* sp.	Mycangimycin (**57**), Frontalamide A (**58**), and Frontalamide B (**59**)	Antimalarial	[87,88]
	Ambrosia beetle, *Fusarium* sp.	Cerulenin (**60**), Helvolic acid (**61**)	Antifungal	[89]
	Rove beetle, *Pseudomonas* sp. [49,50,51,52]	Pederin (**62**)	Anticancer	[90]
	Dung beetle	Tripartilactam (**63**)	Na^+^/K^+^ ATPase inhibitor	[91,92,93,94,95]
	Actinobacteria			
		Coprismycin A–B (**64**–**65**)	Neuroprotective effects	
		Collismycin A (**66**)		
		SF2738D (**67**)		
		Tripartin (**68**)	Histone H3 lysine 9 demethylase KDM4 inhibitor	
	*Streptomyces* sp.	Coprisamides A–B (**69**–**70**)	Quinone reductase inducer	
		Coprisidin A (**71**)	Na^+^/K^+^ ATPase inhibitor	
		Coprisidin B (**72**)	NAD(P)H:quinone oxidoreductase 1 inducer	
	Dung beetle, *Brevibacillus* sp. PTH23	Lenzimycins A–B (**73**–**74**)	Antibacterial	[96]
	*Catharsius molossus*	Molossusamides A–C (**75**–**77**)	-	[97]
Caterpillars	*Helicoverpa armigera*, *Mythimna separata*, *Spodoptera litura*, and *Agrius convolvuli*	Volicitin (80)	-	[98]
		*N*-(17-hydroxy-linoleoyl)-l-glutamine (**81**),		
		*N*-linolenoyl-l-glutamine (**82**),		
		*N*-linoleoyl-l-glutamine (**83**),		
		linolenic acid (**85**), and 17-Hydroxylinolenic acid (**86**)		
Crickets	Infected with *Photorhabdus asymbiotica*	Glidobactin A (**87**)	-	[99]
		Luminmycin A (**88**)	-	
		Luminmycin D (**89**)	Cytotoxic, Proteasome inhibitor	
